# The effect of weather and temporal factors on emergency hand trauma consultations

**DOI:** 10.1007/s00402-023-04777-2

**Published:** 2023-01-27

**Authors:** Claudius Illg, Anna Zoldina, Louisa Sarica, Ruth Christine Schäfer, Adrien Daigeler, Sabrina Krauss

**Affiliations:** Department of Hand, Plastic, Reconstructive and Burn Surgery, BG Unfallklinik Tuebingen, Eberhard Karls University Tuebingen, Schnarrenbergstrasse 95, 72076 Tübingen, Germany

**Keywords:** Hand trauma, Seasonality, Weather, Occupational injury, Gender, External factors

## Abstract

**Introduction:**

Fluctuations in the numbers of patient consultations in hand trauma emergency units are challenging in terms of both scheduling and the provision of sufficient resources. Trauma consultations in general are affected by both temporal and meteorological variables. As the genesis and epidemiology of hand trauma have their own characteristics, this study aimed to identify the influence of temporal and meteorological factors on hand trauma consultations.

**Materials and methods:**

All patients treated for hand trauma in our level one trauma center in 2019 were included in the study population and the data were analyzed in retrospect. The daily weather data, including temperature, sunshine duration, precipitation, humidity and wind speed, as well as temporal factors such as time of day, weekday and public holidays were considered and correlated with patient consultations. Gender differences were studied as well.

**Results:**

We included 4787 hand trauma patients (66.4% male, mean age 38.4 ± 19.3 years, 31.7% occupational injuries). Significantly more consultations occurred on Saturdays as compared to weekdays (14.8 ± 0.6, *n* = 52 vs. 13.0 ± 0.2, *n* = 261; *p* = 0.028), and fewer occurred on official holidays (11.8 ± 0.5, *n* = 63 vs. 13.4 ± 0.2, *n* = 302; *p* = 0.0047). We found a significant positive correlation between daily consultations, sunshine duration (*r* = 0.14, *p* = 0.0056) and the mean temperature (*r* = 0.20, *p* < 0.0001); in contrast, a significant negative correlation between daily consultations and humidity (*r* = − 0.17, *p* = 0.001) was observed. Furthermore, fewer consultations were seen on days with precipitation (12.7 ± 0.3, *n* = 219 vs. 13.8 ± 0.3, *n* = 146; *p* = 0.009). The variation was considerably stronger in men.

**Conclusions:**

Hand trauma consultations increased with increasing temperatures, duration of sunshine, and decreasing humidity. Peak admissions were seen on Fridays and Saturdays. These findings can assist in predicting days with peak admissions to allocate resources appropriately.

## Introduction

A major challenge of acute trauma care is providing immediate care for all patients in spite of variability in trauma numbers. It has been shown that general surgery trauma admission and orthopedic trauma admission numbers globally correlate positively with daily sunshine duration and air temperature and negatively with precipitation [1–5]. Hand trauma can range from superficial lacerations to avulsion or amputation injuries. Due to the high density of functional relevant structures and their simultaneously superficial course in the hand, an injury of these functional structures is frequent. While isolated hand injuries are generally not life-threatening, their treatment and rehabilitation is often time-consuming [6]. As compared to general injuries, the impact of hand injuries on quality of life are often worse, as severe impairment of function can result, making daily life a challenge [7]. The trauma causes of hand injuries differ from general orthopedic trauma. While traffic accidents and falls account for up to 60–80% of orthopedic injuries, they play a minor role in causing hand injuries [8–11], of which 65% are caused by machine, cut and crush trauma [10, 12]. Therefore, the proportion of occupational injuries is greater in hand trauma, explaining the epidemiologic shift toward males in the working-age [12, 13].

Several authors have tried to explain the seasonality of trauma admissions. Recreational outdoor activities that could potentially cause injuries are popular on warm, sunny days during the summer months, particularly among children [14], while heavy rainfall is associated with an increase in slippery outdoor conditions and traffic accidents [15], and snow can increase the risk of falls and lower extremity fractures [16, 17]. Pape-Köhler et al. showed an increase of trauma admissions on weekends due to leisure activities [18]. Conversely, occupational injuries, which make up a major part of hand trauma admissions, could decrease on weekends, public holidays, and school holidays [12, 13].

The BG Trauma Center Tuebingen is an interregional level 1 trauma center and part of the University Hospital Tuebingen, including a specialized, certified department for hand trauma surgery. Concerning hand trauma, it provides services to a catchment area of a large part of southwest Germany, covering both urban and rural areas.

This study investigates the relationship between hand trauma admissions and meteorological factors as well as temporal factors like public holidays. The aim of this study is to assist in predicting the workload in hand trauma units so that sufficient resources can be allotted to days with peak admissions.

## Methods

This study was carried out after approval by the local Research Ethics Committee (project number 521/2020BO) in accordance with the Declaration of Helsinki. All hand trauma patients admitted to our level 1 trauma center between January 1st, 2019 and December 31st, 2019 were included and analyzed retrospectively. Non-trauma related hand-based ER admissions (e.g., arthrosis or infections unrelated to trauma) were excluded. Inpatient and outpatient consultations were considered. In our hospital, hand trauma patients are primarily treated by the department for plastic and hand surgery. Patients admitted to the department of orthopedic and trauma surgery were not included. Patients that suffered from forearm fractures without concomitant hand injuries were treated by the department for orthopedic and trauma surgery and were therefore not included. Days were defined as starting at midnight. A period of investigation ending before 2020 was deliberately chosen to avoid the inclusion of the effects of the COVID-19 pandemic and related lockdowns, as they were shown to influence and reduce the numbers of both trauma cases and the proportion of work-related injuries [19, 20].

### Temporal data

The statistically analyzed temporal factors included month of year, day of week for each individual day and for weekdays versus weekends, school days versus school holidays, and time of day.

### Meteorological data

Daily weather data for the corresponding period were obtained from the national climate data centre of the national meteorological service “Deutscher Wetterdienst” (www.dwd.de). The meteorological data were recorded at Stuttgart-Echterdingen, which is 20 km from the trauma center; it provides the nearest complete weather record and is well within the catchment area of the hospital. The data were considered to be representative of the covered geographical area. Daily readings included the mean, maximum, and minimum temperature at 2 m height as well as the minimum temperature at 5 cm above ground (°C), precipitation (mm), precipitation type (rain, snow), sunshine (hours), cloud coverage (okta), relative humidity (%), vapor pressure (hPa), mean wind speed (m/s), and maximum gust wind speed (m/s). The vapor pressure equals the partial pressure of water vapor in the atmosphere and is therefore a measure of the moisture level of the air. Relative humidity is the ratio of vapor pressure to the equilibrium vapor pressure at a given temperature.

### Statistics

For statistical analysis, two-tailed unpaired *t*-tests were performed to analyze differences in means between two samples, one-way ANOVA was applied for multiple comparisons, and for correlation analysis, the two-tailed Pearson’s *r* was calculated using GraphPad Prism v. 7.04 (GraphPad Software, La Jolla California, USA). Figures were designed using Corel Designer X6 (Corel Corporation, Ottawa, Canada). Values are given as mean ± standard error, and findings were considered statistically significant at *p* < 0.05.

## Results

### Patient characteristics

A total of 4787 hand trauma patients (66.4% male) were admitted during the observed period of 365 consecutive days. The average daily hand trauma volume was 13.1 ± 3.9 patients per day with a widespread range between 3 and 25 patients per day. The mean age of the patients was 38.4 ± 19.3 years (range 0–99). The study population was made up of 8.2% pediatric patients with a mean age of 12.0 ± 4.4 years. Handedness was assessed in 1729 patients. The dominant hand was injured in 48.7%, the non-dominant hand in 49.3%, and both hands in 2.0% of cases. 31.7% of injuries were work-related. The mean time between injury and admission was 1.65 days for work-related trauma and 2.91 days for leisure-related trauma. Of the non-work-related hand trauma cases, 13.9% were covered by private health insurance.

### Annual course of admissions

**Fig. 1:**
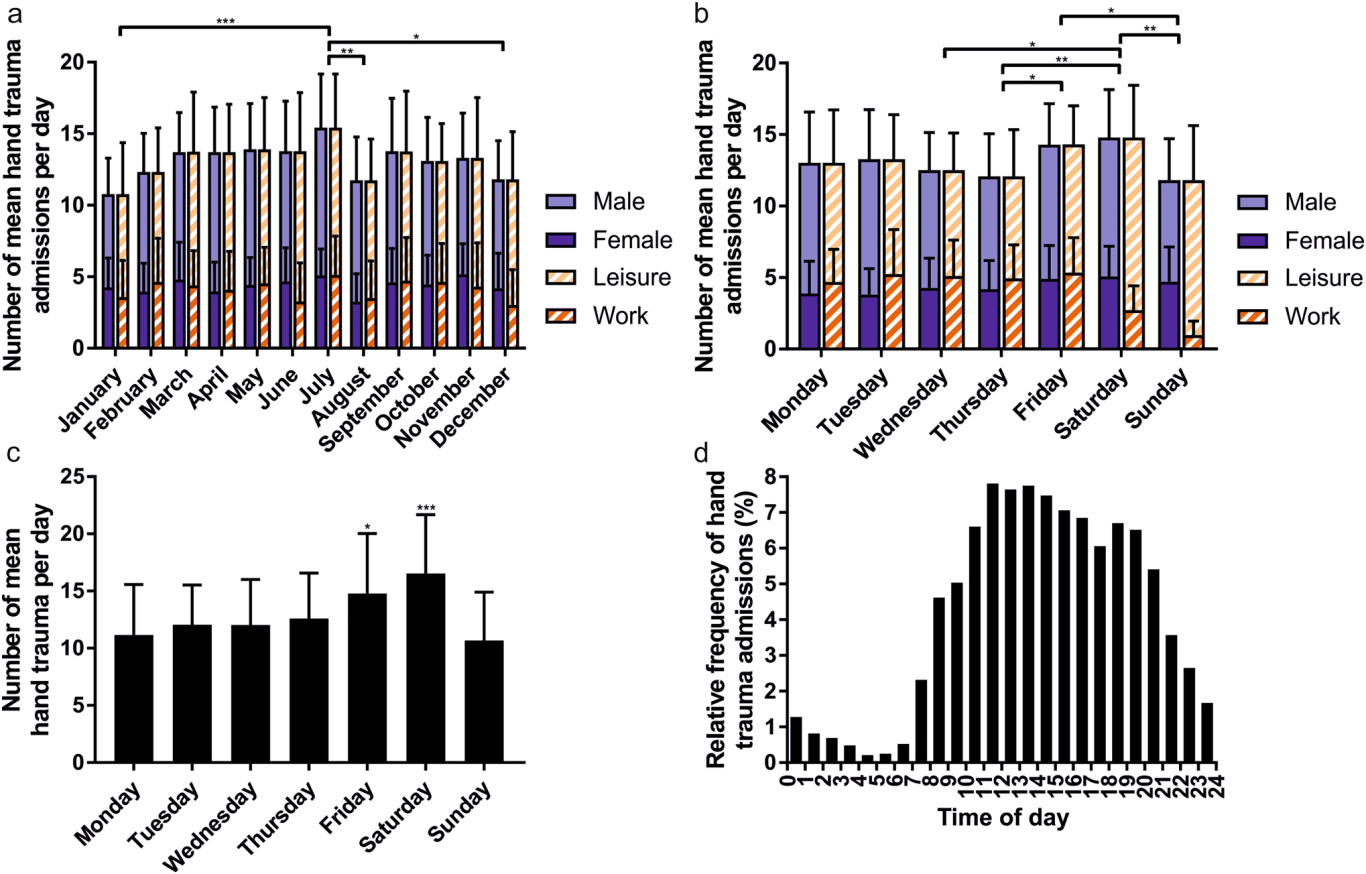
**a** average daily hand trauma volume by month divided by male and female patients as well as work and leisure-related injuries. Significant differences are marked. **b** Average daily hand trauma consultations by day of the week divided by male and female patients as well as work and leisure-related injuries. Significant differences are marked. **c** Average daily hand trauma volume by day of the week; the least significant differences on the peak days Friday and Saturday as compared to other weekdays are shown. **d** The distribution of hand trauma admissions shows a great variation throughout the day. *p < 0.05; **p < 0.01; ***p < 0.0001

Most daily admissions were seen in July, with 15.4 ± 4.2 per day, while the fewest admissions occurred in January (10.8 ± 3.2, *p* = 0.0001), August (11.7 ± 3.5, *p* = 0.009), and December (11.8 ± 3.9, *p* = 0.01). The annual fluctuation of admissions is visualized in Fig. 1a. The proportion of female admissions was significantly higher in January (40.0 ± 17.0%) as compared to August (27.4 ± 14.1%) (*p* = 0.026). The proportion of work-related traumas did not significantly depend on the respective month.

### Weekdays and holidays

Figure 1b illustrates the numbers of hand trauma consultations on different weekdays. When comparing weekdays and weekends, we found significantly more admissions on Saturdays as compared to weekdays (14.8 ± 0.6, *n* = 52 vs. 13.0 ± 0.2, *n* = 261; *p* = 0.028) and significantly less admissions on Sundays as compared to weekdays (11.8 ± 0.6, *n* = 52 vs. 13.0 ± 0.2, *n* = 261; *p* = 0.028). As compared with female patient admissions, there was a greater variation in the rates of male patient admissions throughout the week. The proportion of female admissions was significantly lower on Mondays (28.8 ± 14.5%) and Tuesdays (29.1 ± 12.8%) as compared to Sundays (39.3 ± 15.5%; *p* = 0.0036 and 0.0047). The proportion of work-related hand trauma was significantly lower on Saturdays (18.4 ± 10.9%) and Sundays (8.4 ± 8.4%) as compared to Mondays (36.4 ± 15.7%), Tuesdays (38.7 ± 18.9%), Wednesdays (41.1 ± 18.3%), Thursdays (41.8 ± 18.9%), Fridays (37.1 ± 14.7%) (*p* < 0.0001 each), and on Sundays vs. Saturdays (*p* = 0.019). As for the consultations, the weekdays of injury peaked on Fridays (14.8 ± 5.3) and Saturdays (16.5 ± 5.2) and were lowest on Sundays (10.7 ± 4.2) (Fig. 1c).

The comparison of admissions on Sundays and official public holidays with admissions on all other days showed a pattern of fewer admissions on official holidays (11.8 ± 0.5, *n* = 63 vs. 13.4 ± 0.2, *n* = 302; *p* = 0.0047). Comparing the number of admissions on school holidays with those on all other days showed fewer admissions on school holidays, though the results were not statistically significant (12.5 ± 0.4, *n* = 104 vs. 13.4 ± 0.2, *n* = 261; *p* = 0.06). The proportion of work-related hand trauma admissions was significantly lower during school holidays (23.2 ± 17.6% vs. 35.1 ± 19.3%; *p* < 0.0001).

### Time of day of admission

54.0% of all hand trauma patients were admitted during regular working hours (between 8 a.m. and 4 p.m.) while only 6.6% of all patients were admitted between midnight and 8 a.m. Figure 1d illustrates the hourly distribution of admissions. A peak of admissions is encountered at noon, and then admissions slowly decrease before rapidly dropping after 9 p.m. On weekends, proportional admissions during the night-time between midnight and 6 a.m. were twice as high as compared to weekdays (5.8% vs. 2.9%).

### Sunshine duration

**Fig. 2:**
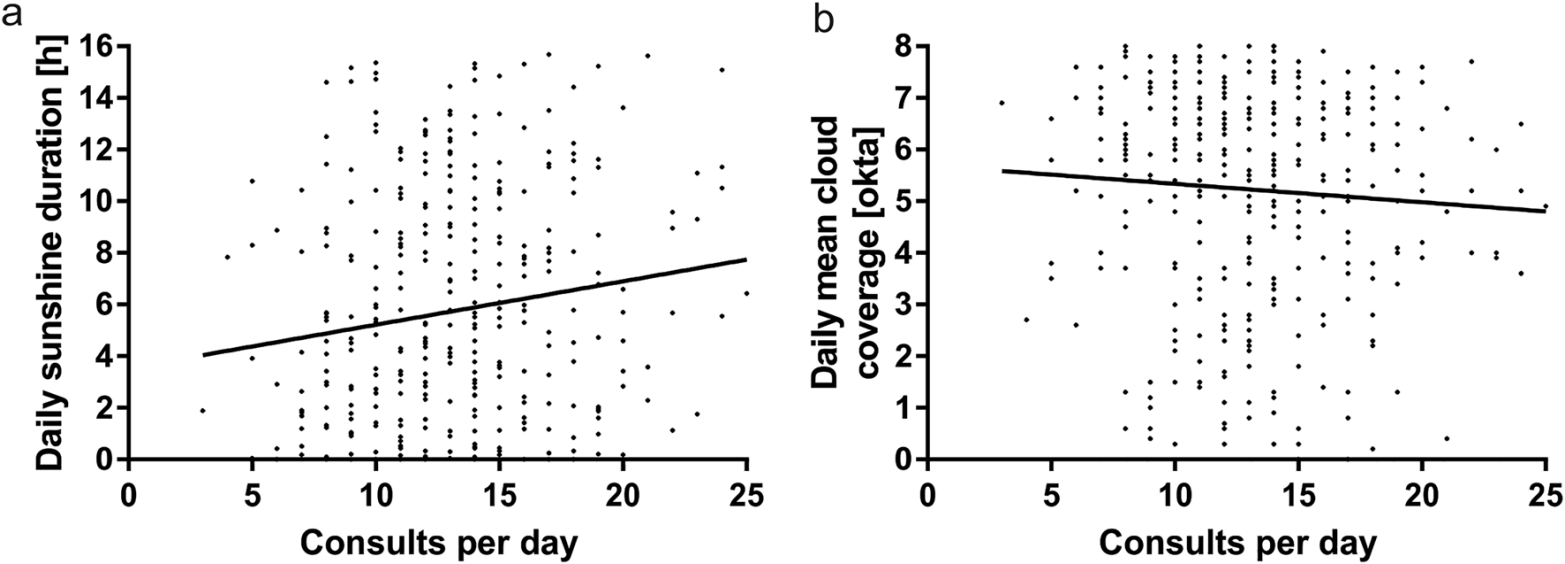
Scatter plot and linear regression lines illustrate the correlation between the number of consultations per day and the daily sunshine duration (**a**) and the daily mean cloud coverage (**b**)

We found a significant positive correlation between daily sunshine duration and numbers of consultations per day (*r* = 0.14, *p* = 0.0056); the results are shown in Fig. 2. Accordingly, daily cloud coverage showed a negative correlation with the number of consultations per day, though it was not significant (*r* = − 0.07, *p* = 0.2). The proportion of female patients was negatively correlated with daily sunshine duration (*r* = − 0.12, *p* = 0.02).

### Precipitation and humidity

**Fig. 3:**
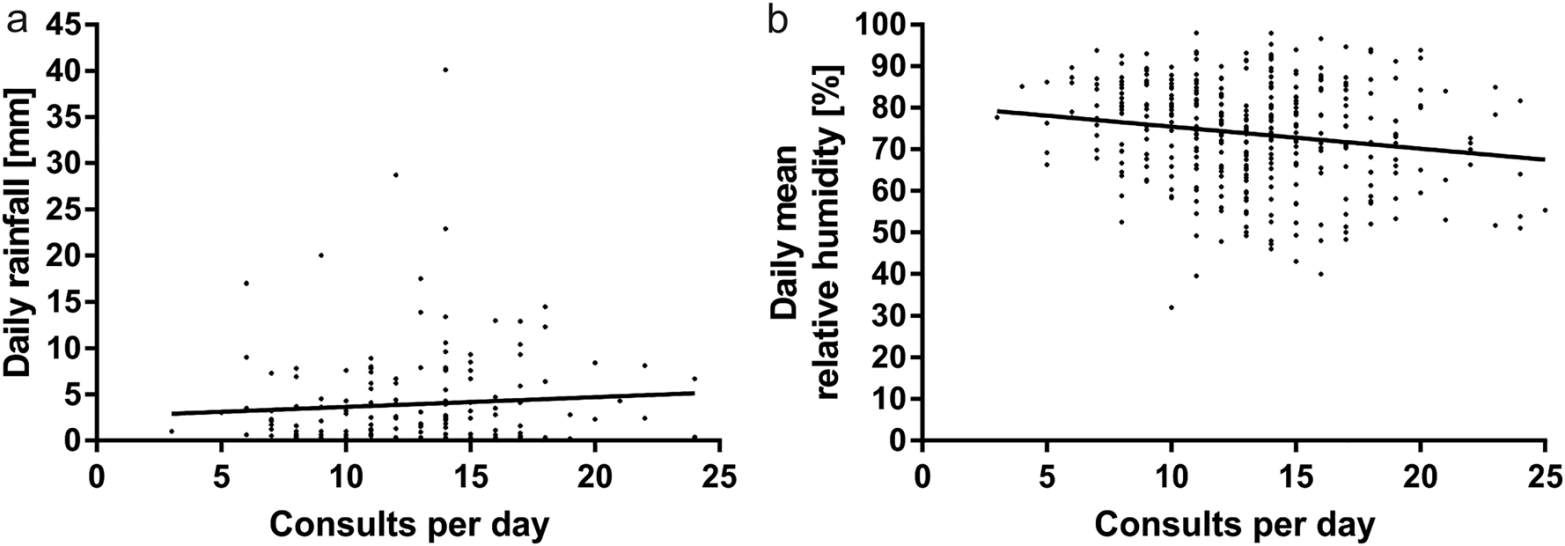
The daily amount of rainfall does not correlate significantly with the daily number of consultations (**a**) (*n* = 219). **b** Illustrates the negative correlation between the mean relative humidity and the number of consultations per day (*n* = 365)

Figure 3 shows scatter plots of rainfall and humidity over daily consultations. We found significantly fewer admissions on days with precipitation (12.7 ± 0.3, *n* = 219 vs. 13.8 ± 0.3, *n* = 146; *p* = 0.009), though the amount of rainfall per day did not correlate significantly with the number of consultations per day (*r* = 0.08, *p* = 0.3, *n* = 168). The daily mean relative humidity showed a significant negative correlation with daily consultations (*r* = − 0.17, *p* = 0.001), while the proportion of female patients correlated positively with the daily mean relative humidity (*r* = 0.11, *p* = 0.03).

### Temperature

**Fig. 4:**
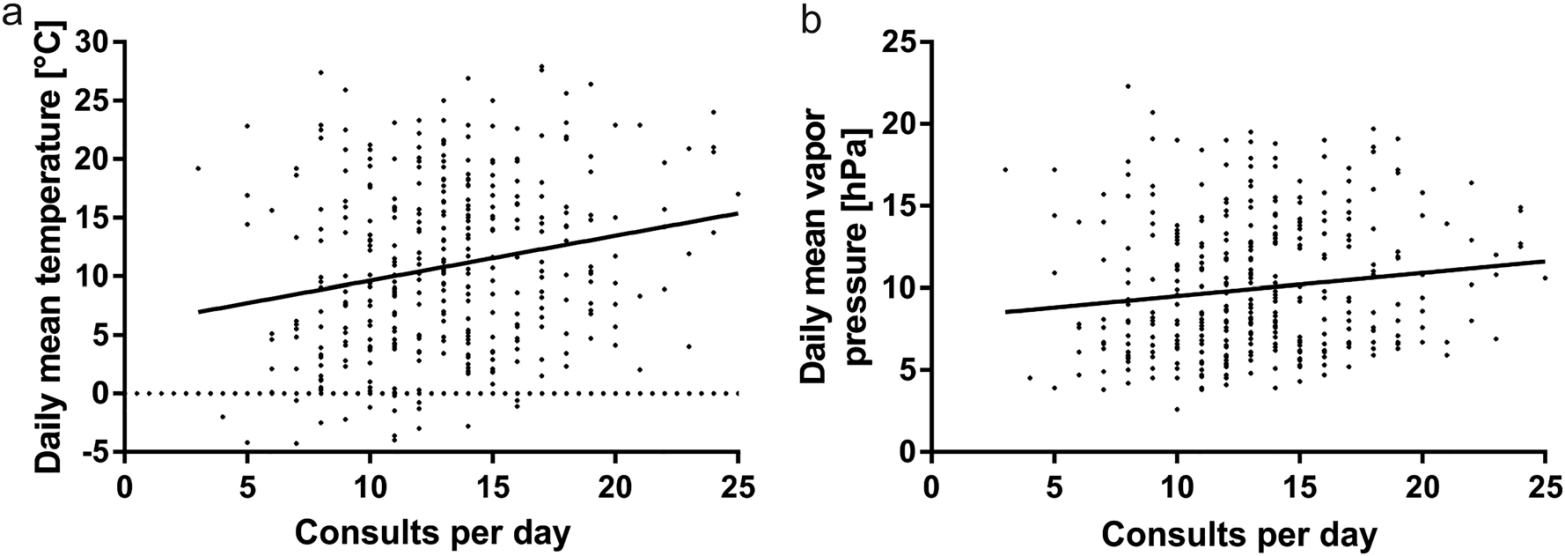
**a** Illustrates the correlation between the mean temperature and the number of consultations per day. **b** illustrates the correlation between the mean vapor pressure and the number of consultations per day

The mean daily temperature correlated positively with the number of daily consultations (*r* = 0.20, *p* < 0.0001), as shown in Fig. 4. Furthermore, the maximum daily temperature, the minimum daily temperature, and the daily minimum temperature at 5 cm above the ground were all positively correlated with the daily consultations (*r* = 0.22, p < 0.0001; *r* = 0.16, *p* = 0.0028; *r* = 0.13, *p* = 0.013). The proportion of female patients correlated negatively with the daily mean temperature (*r* = – 0.11, *p* = 0.038), the daily maximum temperature (*r* = – 0.13, *p* = 0.01), and the daily minimum temperature (*r* = – 0.12, *p* = 0.02). Vapor pressure increases with increasing temperature, and showed a significant positive correlation with the number of consultations per day (*r* = 0.13, *p* = 0.01). The mean daily air pressure showed no correlation with the number of consultations (*r* = – 0.0017, *p* = 0.97).

The proportion of work-related injuries was negatively correlated with the vapor pressure (*r* = – 0.12, *p* = 0.018), the daily mean temperature (*r* = – 0.13, *p* = 0.014), the daily minimum temperature (*r* = – 0.12, *p* = 0.033), and the daily minimum temperature at 5 cm from the surface (*r* = – 0.12, *p* = 0.028).

### Wind

Neither the daily mean wind speed nor the maximum wind speed showed any correlation with the number of consultations (*r* = – 0.032, *p* = 0.54 and *r* = 0.043, *p* = 0.41).

### Injury pattern

**Fig. 5:**
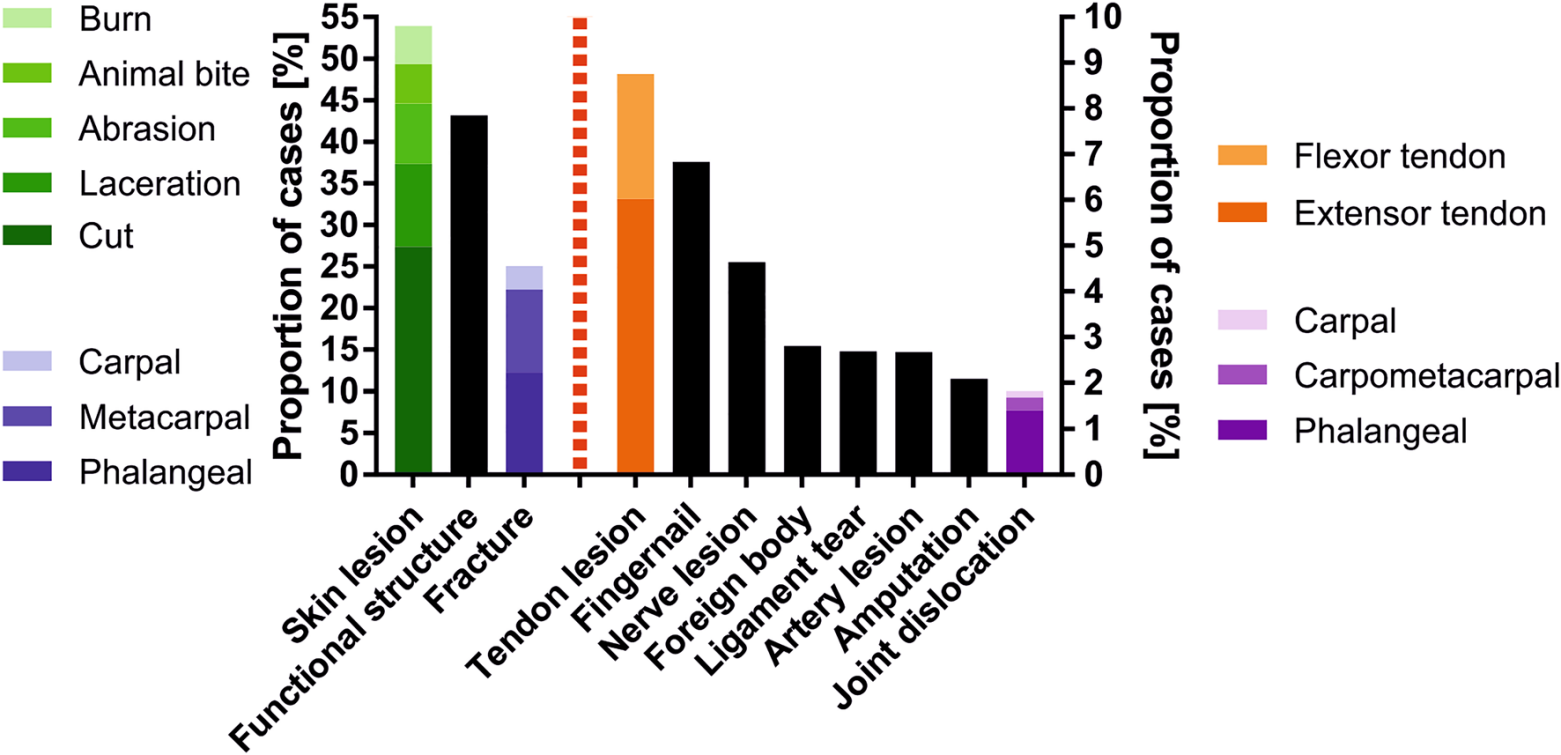
The patients’ injury pattern is illustrated in terms of percentages of cases of the whole study cohort. The dotted red vertical line separates two Y-axes to allow for a more detailed illustration. Injured structures are sorted by frequency in descending order

The patients’ injury pattern is shown in Fig. 5. In 56.8% of the hand trauma patients that sought consultation at our department, we found no injuries to functional relevant structures that required specialized hand surgical expertise. 49.2% of trauma were penetrating; of these, most were due to cuts, followed by lacerations, abrasions, animal bites, and burns. The fracture incidence in our collective was 25.1%, and the incidence of fractures and joint dislocations decreased toward proximal.

Extensor tendon injuries occurred in 6.0% of cases, more than twice as often as flexor tendon injuries (2.7%). Injuries to the fingernail, the nail bed, and associated structures occurred in 6.8% of cases.

## Discussion

Two thirds of all hand trauma patients were male, and approximately one third of injuries were due to occupational accidents; these patients tended to seek treatment earlier. The prevalence of male hand trauma patients has been previously described by Giustini et al., Wu et al., and Dębski and Noszczyk in various populations [21–23]. Men are more often exposed to physical occupational hazards both within and across occupations. The difference within occupations is most likely due to men and women performing different tasks within the same occupation [24]. Furthermore, gender-based differences in leisure-time activities might also expose men to increased hazards as women tend to be more risk-averse than men when it comes to their physical health [25–27]. The frequency of injuries to the dominant hand was comparable to that of the non-dominant hand; while it is worth noticing that the former aggravates the consequences of the injury and may lead to hand-dominance transfer [28].

When looking at the annual course of admissions, a peak in the summer months is apparent, notably, daily admissions were highest in July and lowest in January and February. Occupational and leisure-related injuries fluctuate similarly. Peaks in trauma admissions in the summer months have been previously observed in Germany, Switzerland, Norway, and the USA [2, 18, 29–32]. Accordingly, the summer peak can be explained by common company holidays in craft enterprises and construction industries during December and January and poorer weather conditions for risky leisure activities. This thesis is supported by the observation of a higher proportion of female admissions during the winter months. It is also worth noting that the number of admissions were low in August as well. Considering the fact that both fewer admissions and a lower proportion of occupational traumas were seen during school holidays, the drop of admissions in August can at least partly be attributed to the summer school holidays, where many parents not only take a leave from work but also leave the country for some weeks. Conversely, Tenías et al. and Flinkkilä et al. showed an increase in hip fractures and distal forearm fractures in the winter months due to an increased risk of falling on slippery surfaces [33, 34]. A corresponding effect in hand injuries was not anticipated, as falls account for a small number of hand trauma cases [10].

As expected, Friday and Saturday were the busiest days [18, 31, 32]. While admissions of leisure-related hand trauma increased, work-related hand trauma admissions decreased on Saturdays and Sundays. The numbers of female patient admissions showed less fluctuation both over the month and over the weekdays as compared to males. This can probably be attributed to choices in occupation and leisure activities, as previously discussed. When considering the days that the injuries occurred as opposed to the days of admission, peaks on Fridays and Saturdays were even more considerable.

As expected, the greatest variation in hand trauma incidence can be seen in the time of day of admission [18, 30, 32]. The fact that 46% of patients present after 4 p.m. results in a large workload for the surgeons on duty. Additional available medical staff is therefore required especially in the time period between 4 p.m. and 9 p.m.

The analysis of meteorological data revealed a positive correlation between daily consultations and the daily sunshine duration, minimum, mean, and maximum daily temperature as well as the vapor pressure. In contrast, a negative correlation was seen between daily consultations and daily cloud coverage as well as relative humidity. Furthermore, fewer patients checked in on days with precipitation. In sum, warm, dry, and sunny weather is associated with more admissions and a corresponding increase in the workload for those treating acute hand trauma.

It is worth mentioning, that especially male patients were susceptible to hand injuries in warm, dry, and sunny weather, while the proportion of female patients showed an opposite behavior. When examining occupational hand injuries, we found a decreasing proportion of occupational injuries in warm weather, while the absolute numbers of work-related hand trauma do not show a correlation in this regard. This, in turn, is consistent with the tendency of males to pursue riskier leisure activities [26, 27]. Stomp et al. found that pediatric injuries are more susceptible to the influence of weather [35], though the weather does not seem to be solely responsible for the seasonality in trauma [31, 36, 37].

An increased workload in acute hand trauma emergency units should be expected on warm, dry, and sunny days, especially when those days are Fridays and Saturdays in the summer months. However, it is worth noting that a vast range of daily consultations was detected in all weather conditions, as illustrated by the scatter plots. Therefore, estimations of the workload of the hand trauma unit on the basis of the weather alone are unreliable. However, the combination of all of the previously mentioned factors can assist in providing sufficient medical staff in hand trauma units during peak periods.

56.8% of patients consulted our department with hand injuries that did not necessarily require treatment by a hand surgery specialist as no functional structures were injured. That phenomenon is a consequence of the fact that the German health care system provides patients with direct, free access to specialized emergency department units around the clock. Furthermore, patients have difficulties evaluating the severity of their own injuries and, while few patients underestimate the severity of their injuries and delay seeking medical attention, many overestimate the severity of their injuries and seek immediate treatment from a specialist [38]. The current tendency in Germany of concentrating hand-injury treatment to few specialized centers [39] might improve clinical outcomes while simultaneously increasing workload in these centers. The mandatory assessment of hand trauma emergency unit consultations by a general practitioner, as proposed by van Gils-van Rooij et al., could serve as a filter and reduce the workload for specialist departments while simultaneously buying specialists more time to treat patients with severe injuries [40].

The injury pattern goes along with the most common causes of hand trauma; more than 50% of injuries were penetrating, primarily by cuts. After fractures, the most commonly injured functional structures were tendons, fingernails and associated structures, and nerves.

Local differences in health care systems and the organization of emergency units limit the transferability of our results. In Germany, the treatment of serious occupational injuries is limited to a few specialized trauma hospitals, applicable to our department. This might lead to an overrepresentation of serious work injuries within our patient collective as compared to the general population.

## Conclusion

Hand trauma consultations depend on both temporal and meteorological variables; this influence is considerably stronger in men. Consultations should be expected to peak on warm, dry, and sunny days in the summer months, especially Fridays and Saturdays, between 8 a.m. and 9 p.m.

## Data Availability

The data presented in this study are available on request from the corresponding author. The data are not publicly available due to privacy and ethical restrictions.
